# A decoy chain deployment method based on SDN and NFV against penetration attack

**DOI:** 10.1371/journal.pone.0189095

**Published:** 2017-12-07

**Authors:** Qi Zhao, Chuanhao Zhang, Zheng Zhao

**Affiliations:** 1 Computer Science and Technology College, Jilin University, Changchun, Jilin, China; 2 Department of Public Security Technology, Railway Police College, Zhengzhou, Henan, China; 3 National Digital Switching System Engineering & Technological R&D Center, Zhengzhou, Henan, China; 4 Department of network engineering, Zhengzhou Science and Technology Institute, Zhengzhou, Henan, China; Victoria University, AUSTRALIA

## Abstract

Penetration attacks are one of the most serious network security threats. However, existing network defense technologies do not have the ability to entirely block the penetration behavior of intruders. Therefore, the network needs additional defenses. In this paper, a decoy chain deployment (DCD) method based on SDN+NFV is proposed to address this problem. This method considers about the security status of networks, and deploys decoy chains with the resource constraints. DCD changes the attack surface of the network and makes it difficult for intruders to discern the current state of the network. Simulation experiments and analyses show that DCD can effectively resist penetration attacks by increasing the time cost and complexity of a penetration attack.

## Introduction

Internet has played an important role in various aspects of society, such as education [1], media [2], payment [3, 4], etc. However, the network security issue is becoming increasingly serious. In recent years, intrusion detection technology has made significant progress [5–7]. However, the current technology is still far from ideal in completely preventing intrusions. With the development of network attack technology and continual appearance of new attack methods, intruders are often able to circumvent security mechanisms and penetrate the network. Especially, the zero-day attacks cannot be defensed effectively.

Zero-day attack is a great challenge for defenders, in which attackers exploit unknown vulnerabilities of their target systems. Effective countermeasures, e.g. patch their systems or configure defense systems, cannot be launched since the defenders have no prior knowledge about the vulnerabilities in zero-day attacks. An example of zero-day attacks is Stuxnet worm [8] in 2010, which exploited four unknown vulnerabilities and compromised industrial control systems without being detected. Therefore, it is very necessary to provide network defenders an additional way to deal with such a risk and ensure the network security.

Penetration attack is a type of attack method that combines various network attack techniques and has explicit intentions, such as obtaining sensitive data, gaining administrative access to the network or destroying the network entirely. A penetration attack has the attribute of gradualness, meaning that it often attacks network nodes one by one until it reaches sensitive targets. Penetration attacks are a kind of severe threat to the network security.

The honeypot [9] is the main defensive method against penetration attacks, which is an active defense technique aiming at cheating attackers. With a honeypot, attackers are lured to attack decoy nodes, such as decoy hosts or network services, and their attack behaviors can be caught [10]. Honeypot technologies include honeypot [11], honeynet [12] and honeytoken [13], in which fake data or forged applications are used for attracting attackers into traps so that attackers’ behaviors can be analyzed and stopped efficiently. However, current honeypot technologies are based on an unrealistic assumption: that the attacks will be stopped as long as attackers are lured into a honeypot. Shakarian et al. [14] proposed a more realistic deception protection method based on moving target defense (MTD) [15]. Rather than stopping the attacks, this method delays the attack time and keeps the probability of a successful attack below a given threshold. However, this method assumes the attackers appear only at a fixed position in the network. In fact, the network can be attacked through multiple positions of the network. What’s more, resource constraints are not considered in this method.

Taken together, the above methods are not ideal to defense penetration attacks for two reasons. Firstly, it is difficult for the traditional methods to deploy honeypots due to unavailability of global view of network. Thus, optimized strategies are not optimal. Secondly, traditional methods deploy strategies statically, which is a simplification of reality. In fact, the real attack defense situations are far more complex, and dynamic deployment of strategy can better protect the sensitive targets in networks. However, taking advantage of the SDN and NFV, the global network view can be accessed, thus dynamic service deployment can be achieved. In SDN+NVF architecture[16–18], decoy nodes can be deployed dynamically and efficiently to confuse penetration attackers and protect the network.

In this paper, a Decoy Chain Deployment (DCD) method based on “SDN+NFV” is proposed. DCD monitors the security state of the network globally based on the SDN controller and deploys decoy chains dynamically under certain resource constraints. DCD considers the fact that multiple attack sources and sensitive targets may exist in the network. Moreover, decoy chain strategies are devised based on a simulated annealing (SA) algorithm to maximize the benefit to the network defense.

## Related work

The honeypot is a type of active defense technique with significant published researches studying it. The Argos honeypot [19] is built based on a virtual machine, which monitors the real guest OS and traces received network data using extended dynamic taint analysis. Thus, penetration attacks can be detected and attack features can be extracted automatically. Kuwatly et al. [20] proposed an adaptive honeypot system in a dynamic network environment where active detection and passive recognition tools are combined. Virtual honeypots are dynamically configured in this method. A highly interactive honeypot was proposed by Wagener et al [21]. This method learns attack behaviors and changes the configuration itself dynamically so that attackers are attracted into honeypots and their attack behaviors are revealed. An intelligent honeynet based on SDN, called HoneyMix, was proposed by Han et al [22] The programmability of SDN is utilized to conduct fine-grained control of the flows and the attacker is replied with the most desirable response. Unlike the methods mentioned above, DCD is designed to delay penetration attacks and reduces the probability of sensitive targets being compromised by intruders.

DCD shares the idea of MTD, which takes advantage of dynamically changing the attack surface of a system and repels the attack due to the difficulty of ascertaining the system’s current state. MTD aims to break down the assumption made by attackers of a static network and improves the security of the system by the variety [23–25]. Inspired by MTD, Jafarian et al. [26] proposed a host IP hopping method OF-RHM under SDN, which reduces the effectiveness of scanning attacks. RRM, a route hopping method, was proposed by Qi Duan et al. [27, 28] and can protect 90% of traffic flow from sniffing. Badishi proposed RPH [29], a random port hopping method, which can repel DDoS attacks by changing the communication port. Double hopping communication (DHC) was proposed in [30] and is able to defend against sniffer attacks by changing multiple network configurations dynamically. All of the above methods consider protecting the network before a certain attack is launched. However, DCD is designed to address situations when the network is suffering penetrations.

Based on MTD, Clark and et al. [31] proposed a defense strategy in which the IP addresses of decoy nodes can be hopped to prevent attackers from identifying decoy nodes. The IP addresses of both decoy nodes and real nodes are randomly renewed over time based on an optimum strategy determined by a formal analysis. The same team also modeled the interaction between the attacker and decoy nodes based on the game theory, and the optimized IP randomization strategy can be obtained by the equilibrium analyzing [32]. Both of the two methods above prevent attackers from detecting protected nodes by complicating the terminal nodes of the network with the addition of decoy nodes. However, in this paper, penetration attacks are prevented by increasing the complexity of the network topology. A similar decoy-based method was proposed in [14]. In this method, the graphical representation of network’s logical layout is analyzed, and both the attack time cost and complexity of attacking have been increased by adding “distraction clusters” in the network. However, this method assumes that the intruder attacks a fixed target at only one position in the network without consideration of resource constraints. Nor does it consider the situation in which the intruders go back into the real network again after they fall into a distraction cluster. In this paper, multiple attack sources and sensitive targets are considered based on “SDN+NFV”. We face a more realistic intruder model: intruders may go back into the network again to penetrate more nodes after they fall into a decoy chain.

## Model building

DCD deploys decoy chains in the network based on the network paradigm of “SDN+NFV”. In SDN, centralized control is adopted, where controller plays a core role. The controller can monitor the security status of the whole network and can find the possible attack sources using instruction detection technology [33, 34], attack trace [35–37] and forensic analysis [38, 39]. Taking advantage of SDN, DCD can deploy decoy chains with the knowledge of global network view and security status. NFV enables dynamic service deployment and rapid service delivery in the network. Combining with NFV, DCD can deploy decoy chains dynamically and efficiently, and deal with dynamic network security risks. DCD deploys decoy chains in the generic servers on the data plane to change the attack surface of the network based on the centralized control of the SDN network.

The intruders might appear on multiple network nodes, as there may be some less protected nodes or multiple potential intruders. And in the network, there are multiple sensitive targets where sensitive data is stored. If one of the sensitive targets is compromised by intruders, the defense of the network fails. Therefore, multiple attack sources and multiple sensitive targets should be considered in the penetration model. In addition, we only consider the penetration attack, where intruders attack networks through the nodes connecting directly.

### The penetration topology model

In SDN, a network is defined as a node set *S* = {*s*_1_, *s*_2_,⋯, *s*_n_}, where *s*_*i*_ can be an access switch, a core switch or a middlebox, etc. The penetration attack can be represented as penetration topology model *Z* = (*S*,*R*,*π*,*f*,*O*,*T*), where *S* is a network system, *R* ∊ *S* × *S* is a directed edge set representing the relations between nodes, *π* is defined as the compromise probability function *S* × *S* → [0,1], or the probability of compromising node *s*′ when the intruder has obtained the controlling authority of node *S*. *π* has two properties as follows.

$$\text{For}\forall (s,s^{\prime })\notin R,\;\pi (s,s^{\prime })=0$$ (1)

$$\text{For}\forall (s,s^{\prime })\in R,\;\pi (s,s^{\prime })>0$$ (2)

Formulas (1) and (2) show that if nodes *s* and *s*′ are not connected directly, intruders cannot penetrate *s*′ from *s* directly, otherwise they can penetrate node *s*′ with a probability greater than zero. Based on existing security risk metric standards of network device (such as CVSS [40]) and attack graph-based probabilistic security metric [41], the probability of each node in the network being compromised can be evaluated.

The sweetness function *f* is defined as *S* × *S* → ℝ^+^, which evaluates the attraction of the node to intruders. The value of *f* (*s*, *s*′) represents the willingness of penetrating node *s*′ with the controlling authority of node *s*. The property of *f* is shown in formula (3). If *s* and *s*′ are not directly connected, the sweetness of *s*′ for *s* is zero.

$$\text{For}\forall (s,s^{\prime })\notin R,\;f(s,s^{\prime })=0$$ (3)

**Fig 1:**
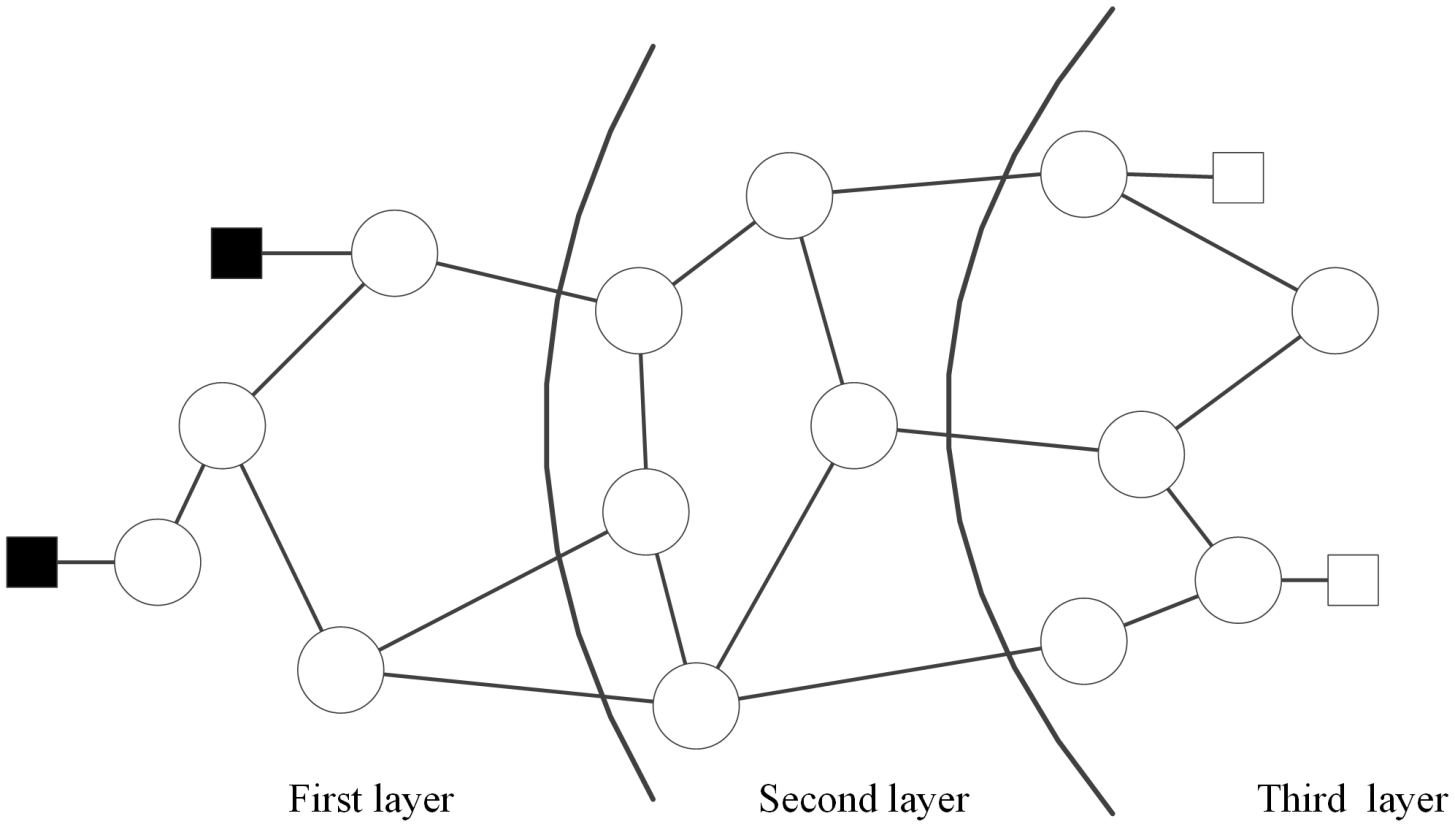
An example of layer structure of penetration topology.

Let *O* and *T* denote the attack source set and sensitive target set, respectively. Intruders start the network attack by attacking nodes from *O* until an arbitrary sensitive target in *T* has been reached. In reality, the network is layered. Let L(s) denote the layer of node *s*. We only consider outside intruders that locates at the edge of network, while the sensitive targets are at the innermost layer of the network. Intruders have to penetrate the network layer by layer to get to a sensitive target. Therefore, the penetration topology model is layered as well. We show an example of penetration topology with 3 layers in Fig 1. The intruders (the solid squares) are present at the first layer of the penetration topology and sensitive targets (the hollow squares) are at the third layer.

**Fig 2:**
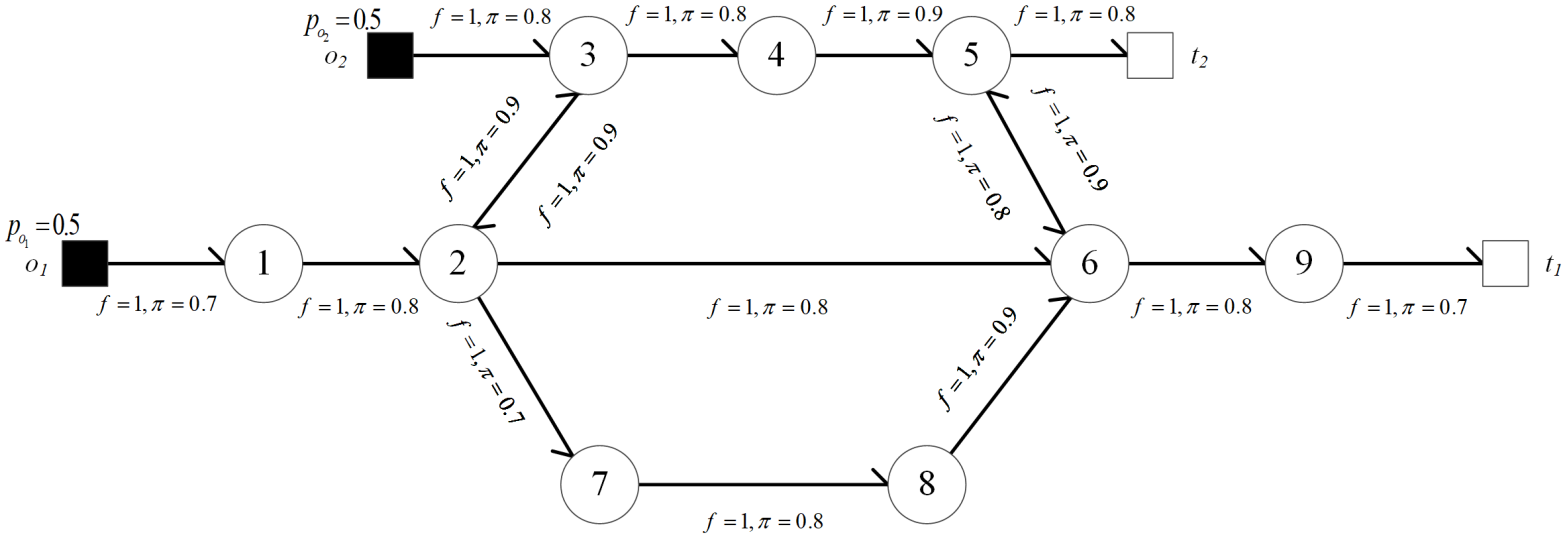
An example of penetration topology. (First layer: node 1, 2, 3; Second layer: node 4, 7, 8; Third layer: node 5, 6, 9).

A penetration topology is represented as a directed graph and an example of penetration topology is shown in Fig 2. In this penetration topology, the attack source set and the sensitive target set are *O* = {*o*_1_, *o*_2_} and *T* = {*t*_1_, *t*_2_}, respectively. Intruders will penetrate the network from the nodes in *O* with identical probability, i.e. $p_{o_{1}}=p_{o_{2}}=0.5$. *π* and *f* are labeled on the directed edges in the penetration topology. The directed edges represent the penetration direction of an attacker.

### The attack model

Devices, such as switches and middleboxes could be attacked, if attackers take advantage of their MAC addresses. Attackers could launch specific attacks taking advantage of the vulnerabilities of the devices by sending some carefully constructed packets to them. Vulnerabilities will be triggered, when these devices process the constructed packets. Thus, these devices will be penetrated. Due to the gradualness of penetration attacks, intruders the penetrate network node by node along the paths in the network’s penetration topology. In this section, the definition of “penetration path” is described. Then, the formalization description of the penetration attack model is presented. Finally, the traceback of the penetration path is shown.

#### Penetration path

The node sequence through which the network is attacked by an intruder is called the penetration path, which is denoted as *σ* and satisfying in formula (4). *ω*(*σ*) represents a set of node pairs where two nodes are attacked successively in *σ*. The length of the penetration path is represented as |*σ|*, which is the number of nodes in *σ*.

$$\left(s,s^{\prime })\in \omega \left(\sigma \right)\to (s,s^{\prime })\in R\wedge L(s)\leq L(s^{\prime }\right)$$ (4)

**Fig 3:**
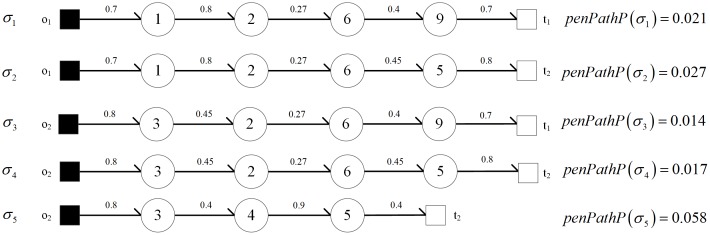
The penetration paths reaching sensitive targets with length less than 5. (The number marked on each directed edge represents the node penetration probability, which is detailed in Section 3.2.2. *penPathP*(*σ*) is path penetration probability, which is described in Section 3.2.2).

For a penetration path *σ*, $next(s,\sigma )=\{s^{\prime }|s\in \sigma \wedge s^{\prime }\notin \sigma \wedge \left(s,s^{\prime }\right)\in R\wedge L(s^{\prime })\geq L(s)\}$ is defined to represent the set of next nodes that can be potentially penetrated at node *s* on penetration path *σ*. In the penetration topology as shown in Fig 2, the penetration paths from *O* to *T* satisfying |*σ|* ≤ 5 are *σ*_1_, *σ*_2_,⋯, *σ*_5_. The penetration paths are shown in Fig 3, where the number marked on each directed edge represents the node penetration probability (probability of one node being penetrated, detailed in Section 3.2.2). At the rightmost of corresponding paths, *penPathP*(*σ*) is path penetration probability (probability of one path being penetrated, described in Section 3.2.2).

#### Penetration attack model

There are limits to an attacker’s abilities due to limited cost he can afford. Moreover, network defense measures, such as intrusion detection, can detect intrusion behaviors. Therefore, the number of nodes that an intruder can penetrate continuously is limited. Let *ξ* denotes the maximum length of the penetration path, representing the ability of an intruder. Bigger *ξ* corresponds to greater ability of an intruder. Suppose that in a penetration topology, *s* is the current node on a penetration path *σ* (*s* ∊ *σ*). The probability of an intruder selecting (*s*′ ∊ *next*(*s*, *σ*) as the next node is *selNodeP*, as shown in formula (5). This formula describes the fact that the intruders tend to select a more attractive node to penetrate.

$$selNodeP\left(s,s^{\prime },\sigma \right)=\frac{f\left(s,s^{\prime }\right)}{\sum_{s^{\prime \prime }\in next(s,\sigma )}f\left(s,s^{\prime \prime }\right)}$$ (5)

It can be assumed that for an intruder, selecting *s*′ as the next node to penetrate is independent with compromising *s*′ successfully. Hence, the probability of selecting *s*′ and compromising *s*′ for an intruder can be represented as *penNodeP*, which can be obtained by formula (6), called node penetration probability.

$$penNodeP\left(s,s^{\prime },\sigma \right)=selNodeP\left(s,s^{\prime },\sigma \right)\times \pi \left(s,s^{\prime }\right)=\frac{f\left(s,s^{\prime }\right)\times \pi \left(s,s^{\prime }\right)}{\sum_{s^{\prime \prime }\in next(s,\sigma )}f\left(s,s^{\prime \prime }\right)}$$ (6)

Given the attack source *s*_0_, an intruder can penetrate the network along penetration path *σ =* 〈*s*_0_, *s*_1_,⋯, *s*_*n*_〉, and reach *s*_*n*_ with probability *penPathP*(*σ*) calculated from formula (7), called the path penetration probability. *p*_*σ*(0)_ denotes the probability that the intruder appears at the first node of *σ* (*σ*(0) ∊ *O*).

$$penPathP\left(\sigma \right)=p_{\sigma (0)}^{}\times \prod_{\left(s,s^{\prime }\right)\in \omega \left(\sigma \right)}^{}penNodeP\left(s,s^{\prime },\sigma \right)$$ (7)

As shown in Fig 3, the path penetration probabilities of *σ*_1_ ~ *σ*_5_ can be calculated based on formula (7). In the penetration topology as shown in Fig 2, the intruder successfully penetrates one node of *T* from *O* and satisfies |*σ*| ≤ 5 with probability $\sum_{i\in [1,5]}penPathP\left(\sigma_{i}\right)=0.137$.

#### Traceback of penetration path

An intruder launches a penetration attack from the first layer of the network, then he penetrates the network along the directly connected nodes. The layers of network are public information. The intruder knows the layer he is currently at and always selects a node to penetrate that is at the same or higher layer of the network. If all the nodes directly connected to the current penetrated node have already been compromised or the layers they belong to are lower than the current layer, the intruder will trace back along the penetration path and continue to penetrate the network through the first node with penetrable neighbor nodes.

**Fig 4:**
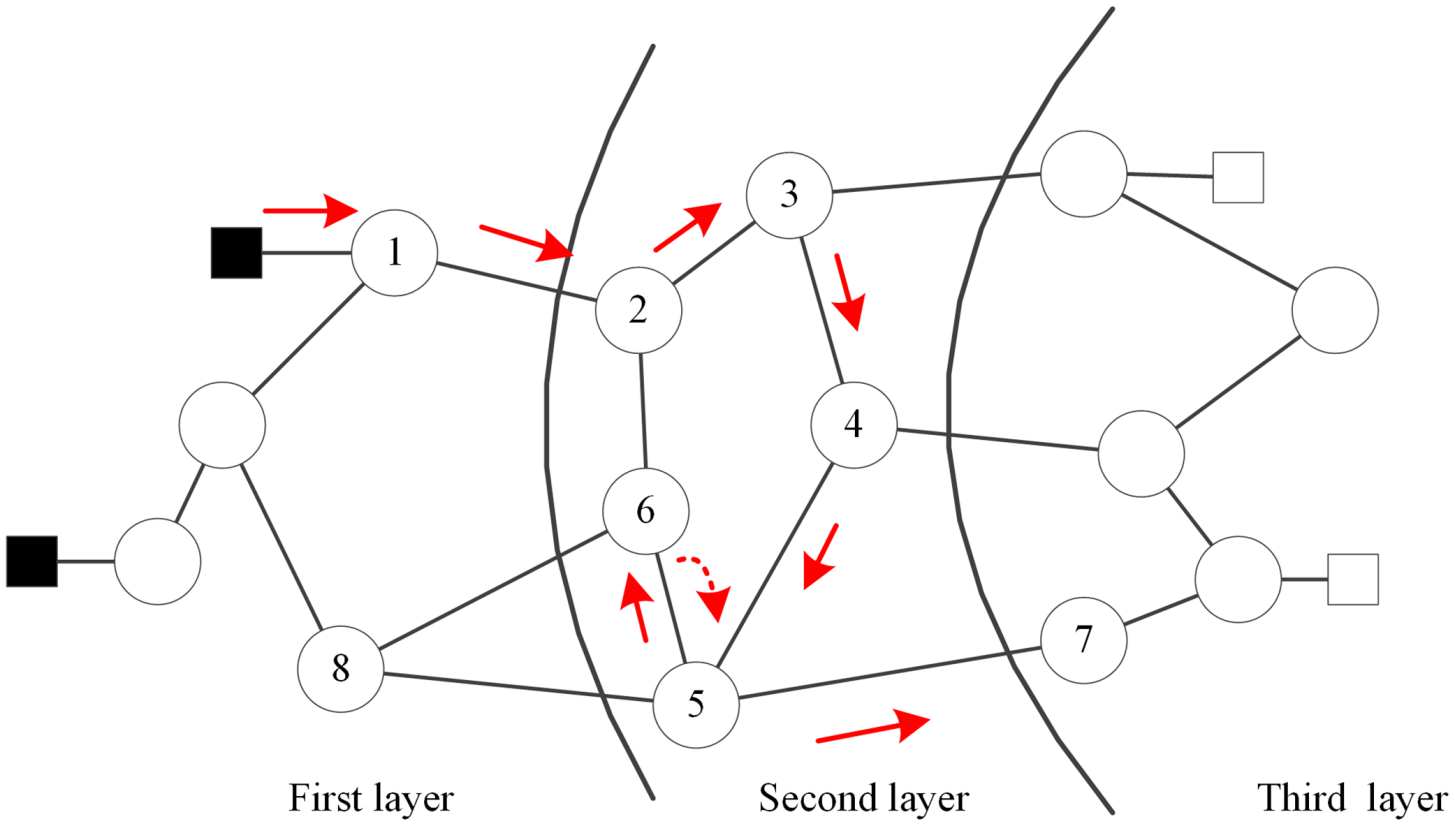
An example of traceback of the penetration path.

Fig 4 shows an example of traceback of penetration path in a 3-layer network. The intruder starts from the first layer of the penetration topology and launches a penetration attack along the path *σ* = 〈1, 2, 3, 4, 5, 6〉. When the intruder continues to penetrate at node 6, he has to trace back to node 5 to find a new node to penetrate, since all the neighbor nodes except node 8, which is at lower layer, have been compromised. The uncompromised neighbors of node 5 include node 7 and 8. The intruder selects node 7 as the next target to penetrate because the layer of node 8 is lower than node 5. Then, the penetration path is updated with *σ*′ = 〈1, 2, 3, 4, 5, 6, 7〉 and (5,7) ∊ *ω* (*σ*′). The traceback of the penetration path described here is a realistic assumption of penetration attack.

### Decoy chain model

A decoy chain is a one-way sequence of virtual machines that can function as decoy switches, middleboxes or terminal hosts. Once the intruder falls into a decoy chain, he will continue to penetrate the decoy nodes along the decoy chain. Decoy chains are different from traditional honeypots, as they do not stop intruders from attacking the network. Instead, intruders will be attracted into decoy chains so that their attacks are delayed, and the probability of sensitive targets being penetrated is decreased. The decoy chain is defined as *dc* = (*id*, Π, *F*, *l*), where *id* is the unique identification of a decoy chain. Π is a set of compromised probabilities for the nodes in the decoy chain and *F* consists the sweetnesses for them. *l* is length of the decoy chain, or the number of virtual machines in the decoy chain.

There are 3 decoy chains in decoy chain set *DC* = {*dc*_1,_ *dc*_2,_ *dc*_3_}, and their configurations are shown as follow.

$$dc_{1}=\{\begin{array}{c} \forall \pi \in \Pi ,\pi =0.9\\ \forall f\in F,f=1\\ l=3 \end{array}\;\;dc_{2}=\{\begin{array}{c} \forall \pi \in \Pi ,\pi =0.9\\ \forall f\in F,f=1\\ l=4 \end{array}\;\;dc_{3}=\{\begin{array}{c} \forall \pi \in \Pi ,\pi =0.8\\ \forall f\in F,f=1\\ l=5 \end{array}$$

**Fig 5:**
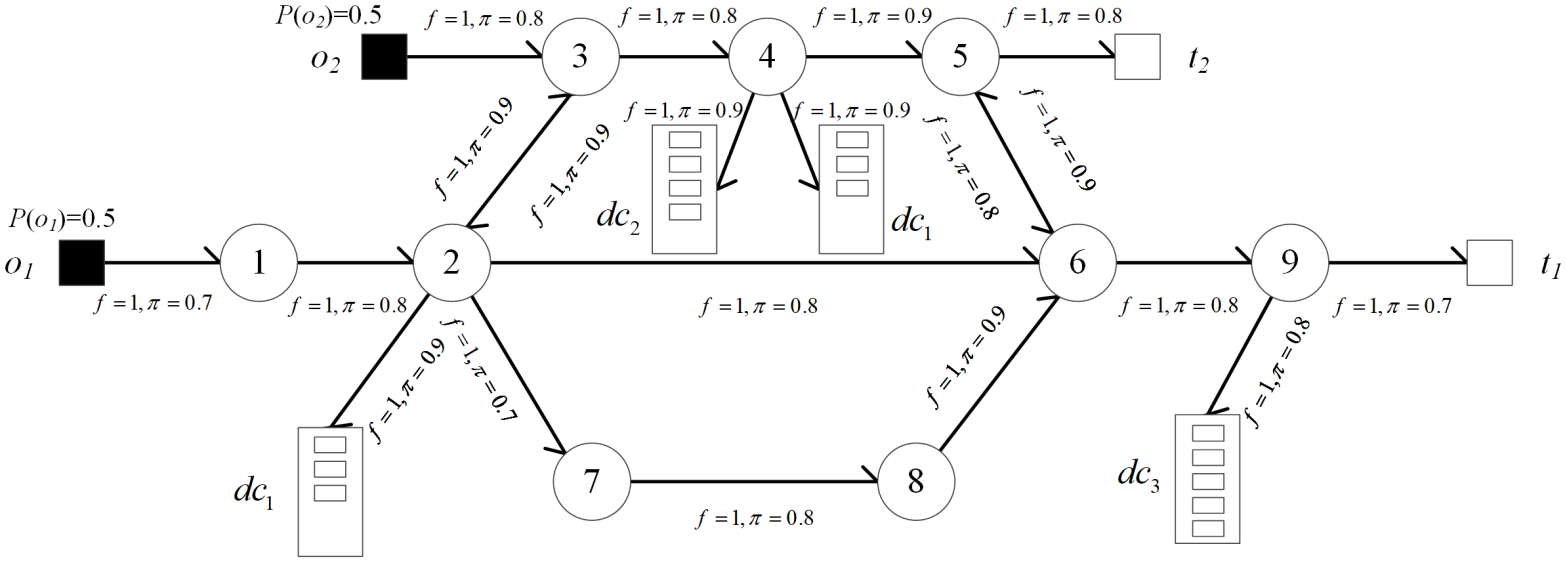
The penetration topology deployed with decoy chains.

The length of *dc*_1,_ *dc*_2_ and *dc*_3_ are 3, 4 and 5, respectively, and all their sweetnesses are 1. Multiple instances of each decoy chain can be deployed in the network. For the penetration topology in Fig 2, one decoy chain’s deployment strategy is shown in Fig 5. In the penetration topology with this decoy chain deployment strategy, the intruder successfully penetrates one node of *T* from *O* and satisfies |*σ*| ≤ 5 with probability $\sum_{i\in [1,5]}penPathP\left(\sigma_{i}\right)=\;0.0781$. The probability is decreased by 43% comparing to the original network shown in Fig 2.

## Decoy chain deployment

Within the framework of SDN+NFV, DCD utilizes the server resource to deploy decoy chains under centralized management of the controller. To avoid an intruder penetrating two identical decoy chain instances from one node, we assume that a decoy chain cannot be deployed in one server more than once. Given resource constraints, optimizing the deployment of decoy chains to maximize the benefit to the network defense is a DCD problem.

### Decoy chain deployment model

Let *Z* denote the penetration topology of a SDN network *S*. Define *V* = {*v*_1_, ⋯, *v*_n_} to denote the servers in the data plane, where one or more virtual machines can run. Assume every forwarding node in *Z* connects with a server, i.e., for ∀*s*_*i*_ ∊ *Z*, ∃*v*_i_ ∊ *V* ˄ (*v*_i_ connects with *s*_*i)*_. For potential attack sources and sensitive targets, the SDN controller generates the deployment strategies and deploys decoy chains in the data plane for minimizing the probability that sensitive targets are compromised. Let *DC* denote the set of decoy chains that are available to defender. Variable $x=\left\{x_{i}^{j}|i\in [1,\left|V\right|],j\in [1,\left|DC\right|]\right\}$ denotes one deployment strategy where $x_{i}^{j}\in \{0,1\}$. $x_{i}^{j}=1$ indicates that an instance of *dc*_*j*_ ∊ *DC* is deployed in server *v*_*i*_. Otherwise, no instance of *dc*_*j*_ is deployed in *v*_*i*_. For *Z*, the DCD problem is to minimize formula (8), where *Z*^+^ represents the penetration topology deployed with strategy *x*. Penetration probability $P_{Z^{+}}^{O,T}\left(x\right)$ is the probability that an intruder reaches *T* from *O* under the condition of *Z*^+^.

$$\text{Min}\;DCD\left(x\right)=P_{Z^{+}}^{O,T}\left(x\right)$$ (8)

Under penetration topology *Z*^+^, the probability of reaching a sensitive target satisfying |*σ*| ≤ *ξ* is shown in formula (9). $Path_{Z^{+}}^{O,T}\left(x,\xi \right)$ represents the set of penetration paths in *Z*^+^ from *O* to *T* that have a length no bigger than *ξ*.

$$P_{Z^{+}}^{O,T}\left(x\right)=\sum_{\sigma \in Path_{Z^{+}}^{O,T}\left(x,\xi \right)}penPathP\left(\sigma \right)$$ (9)

Due to limited resources, such as number of CPU, memory size, HDD and bandwidth, the number of virtual machines running on a server are limited. In this paper, the resource constraints are simplified and the capacity of a server is used to represent all the resource provided by a server. The capacity of a server is defined to be the maximum number of virtual machines that could run on it at the same time. For a server *v*_*i*_ ∊ *V*, its capacity is denoted as *c*_*i*_; in other words, the number of virtual machines running on *v*_*i*_ is no more than *c*_*i*_. The server capacity constraint in DCD is shown in formula (10), where *dc*_*j*_ ∊ *DC* and *l*_*j*_ denotes the number of virtual machines in *dc*_*j*_.

$$\text{For}\forall i\in [1,\left|V\right|],\;\sum_{j\in [1,\left|DC\right|]}l_{j}\times x_{i}^{j}\leq c_{i}$$ (10)

**Fig 6:**
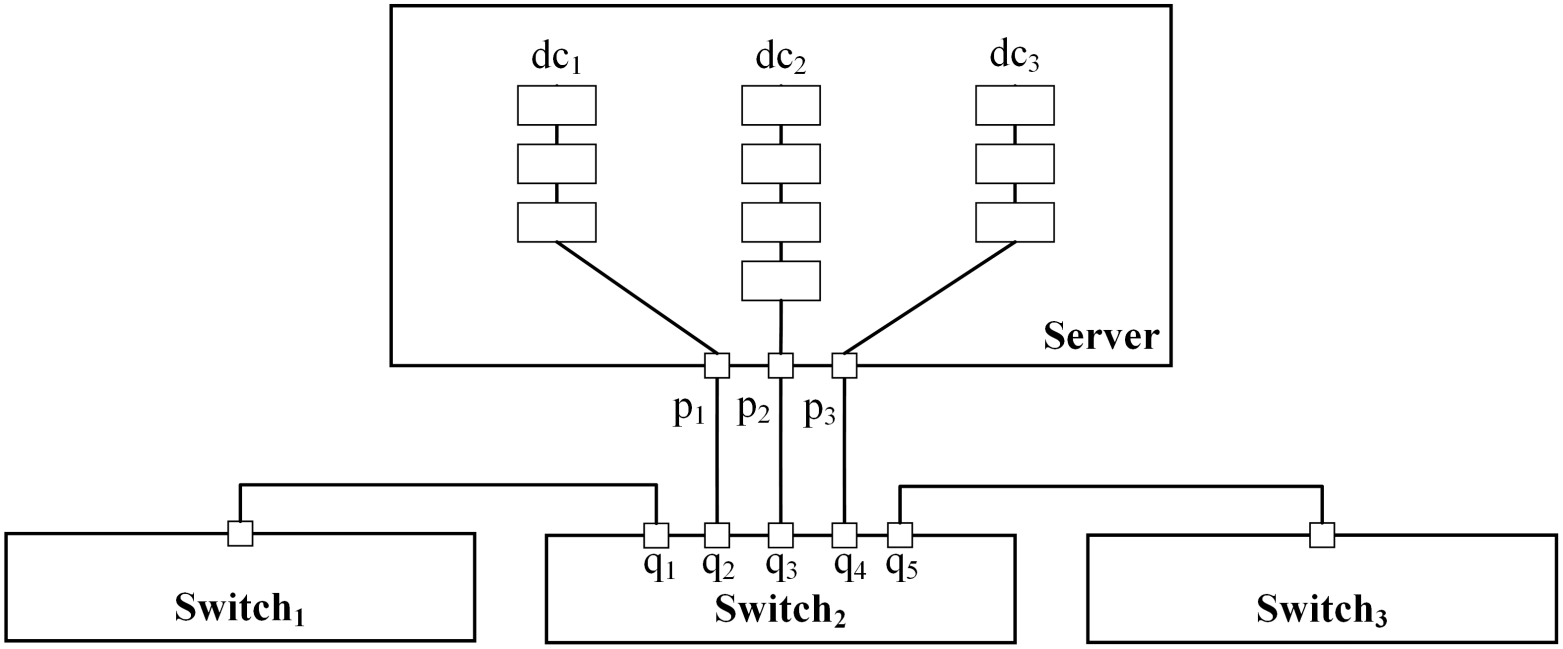
The connection between one server and one switch.

When multiple decoy chain instances are deployed in a single server, each decoy chain needs to occupy one port connecting to an SDN switch to simulate a real network branch, as shown in Fig 6. As the number of ports on servers and switches are limited, the number of decoy chains that a server can load is limited as well. For example, the load of decoy chains of the server in Fig 6 is less than 3. Given the maximum load *h*_*i*_ of server *v*_*i*_, DCD satisfies the constraint as shown in formula (11).

$$\text{For}\forall i\in [1,\left|V\right|],\sum_{j\in [1,\left|DC\right|]}x_{i}^{j}\leq h_{i}$$ (11)

In the servers on data plan, other service functions also need to be deployed, such as firewalls and traffic monitors. Therefore, the total capacity that decoy chains can occupy is limited. Suppose that *θ* is the maximum service capacity utilization rate, then the capacity occupied by decoy chains satisfies the constraint as shown in formula (12).

$$\sum_{i\in [1,V]}\sum_{j\in [1,\left|DC\right|]}x_{i}^{j}\times l_{j}\leq \theta \sum_{k\in [1,\left|V\right|]}h_{k}$$ (12)

### The solution algorithm of DCD problem

Decoy chain deployment is similar to the server chain deployment [42–44], but their targets are different. The target of DCD is to decrease the penetration probability from one attack source node to the sensitive target set. The solution to DCD problem is to find the most optimal position for decoy chains and to maximize the benefit to the network defense under certain resource constraints. An SA algorithm is utilized in this paper to solve the problem.

#### Penetration probability computation

For a decoy chain deployment strategy *x*, an intruder could select any attack source to launch a penetration attack. Algorithm *PenetrateProbability* computes the probability that the intruder reaches a sensitive target in *T* from *O* (see Algorithm 1). In this algorithm, line (2) loops for each attack source o_*i*_ ∊ *O* to get penetration probability from *o*_*i*_ to any sensitive target in *T*. Line (3) of the algorithm computes the probability that the intruder reaches a sensitive target in *T* from one attack source *o*_*i*_. $p_{o_{i}}^{0}$ is the probability that the intruder is present at attack source *o*_*i*_.

**Algorithm 1:** Penetrate probability from attack source set to sensitive target set.

**Input**: *Z*^+^,*O*,*T*,*ξ*
**Output:$P_{Z^{+}}^{O,T}$**
*PenetrateProbability*(Z^+^,*O*,*T*,*ξ*)
(01)*P* = 0
(02)**for** *o*_*i*_ **in** *O*
(03) $P\leftarrow P+\;p_{o_{i}}^{0}\times$*RecursionTraversing* (*Z*^+^,*T*,*ξ*,*o*_*i*_,Ø,1,0)
(04)**return** *P*

*RecursionTraversing* is a recursion algorithm that uses the depth-first search method to compute the penetration probability on each path from an attack source to a sensitive target set, as shown in Algorithm 2. *RecursionTraversing* starts the recursion from node *s*. If the current node is a sensitive target, it adds the penetration probability to variable *sum* and returns (lines (1)~(3)). Otherwise, it makes recursive call (line (4)~(17)). Firstly, a neighbor node of *s* in *next*(*s*, *σ*) is selected (line (6)). The path is then updated, the penetration probability of the new path is computed and the recursive call is made (line (7)~(9)). If *next*(*s*, *σ*) = Ø, it traces back *σ* to find a node *s*′ that satisfies *next*(*s*′, *σ*) ≠ Ø (line (11)~(15)). The traceback will be stopped if a sensitive target is reached (line (16)~(17)).

**Algorithm 2:** Penetration probability from one attack source node to sensitive target set using recursion traversing.

**Input**: *Z*,*T*,*ξ*,*s*,*σ*,*p*, *sum*
*Z*: Penetration topology;
*T*: Sensitive target set;
*ξ* Maximum length of penetration path;
*s*: An attack source node;
*σ*: Current penetration path;
*p*: Probability of a penetration path;
*sum*: Probability of all the penetration paths from *s* to *T*;
**Output:$P_{Z}^{s,T}$**
$P_{Z}^{s,T}$ is the penetration probability from *s* to *T* in penetration topology *Z* within max length *ξ*.
*RecursionTraversing* (*Z*,*T*,*ξ*,*s*,*σ*,*p*,*sum*)
(01)**If** *s* ∊ *T* //For *s* is a sensitive target
(02) *sum← sum* + *p*
(03) **return** *sum*
(04)**else if** |*σ*| < *ξ* //For *s* is not a sensitive target
(05) **if** *next*(*s*, *σ*) ≠ Ø
(06) **for** *s*_*i*_ ∊ *next*(*s*, *σ*)
(07) *newσ* ← *σ* ⋃ *s*_*i*_
(08) *newp* ← *p* × *penNodeP*(*s*,*s*_*i*_,*σ*)
(09) *sum* ← *RecursionTraversing*(*Z*^+^,*T*,*ξ*,*s*_*i*_,*newσ*,*newp*,*sum*)
(10) **else**
(11) Loop traceback *σ* to get a node *s*′ **satisfy** *next*(*s*′, *σ*) ≠ Ø
(12) **for** *S*_*j*_ ∊ *next*(*s*′, *σ*)
(13) *newσ* ← *σ* ⋃ *s*_*j*_
(14) *newp* ← *p* × *penNodeP*(*s*′,*s*_*j*_,*σ*)
(15) *sum*←*RecursionTraversing* (*Z*^+^,*T*,*ξ*,*s*_*j*_,*newσ*,*newp*,*sum*)
(16) **if** any sensitive target in *T* is penetrated
(17) **return** *sum*
(18)**return** *sum*

Algorithm *RecursionTraversing* goes over all the paths through which an intruder may potentially attack the network. However, it is less applicable for large scale networks because the number of penetration paths increases exponentially with respect to the size of the network. To solve this problem, DCD uses a random sampling method to select penetration paths. As shown in Algorithm 3, *maxCnt* penetration paths are randomly selected in *RandomSampling*, and the penetration probability of these paths are computed. In general, the larger the size of network is, the bigger value of *maxCnt* should be selected, as there are more penetration paths in a larger network. *RandomSampling* is similar to the algorithm proposed in literature [14]. However, we consider the traceback possibility and the layers of the network, which is more realistic. In *RandomSampling*, a node *s*_*i*_ is selected as the penetrated node from set *next*(*s*, *σ*) with probability *penNodeP*(*s*,*s*_*i*_,*σ*) for simulating the penetration attack (line (6)~(10)). If *next*(*s*, *σ*) = Ø, it traces back along *σ* until a penetrable neighbor node is selected (line (12)~(16)). If we use the random sampling method to evaluate penetrate probability from attack source set *O* to sensitive target set *T*, we need to replace the function *RecursionTraversing* in line (3) of Algorithm 1 with *RandomSampling*, described in Algorithm 3.

**Algorithm 3:** Penetration probability from one attack source node to sensitive target set using random sampling paths.

**Input**: *Z*,*T*,*ξ*,*s*,*σ*,*p*,*sum*
**Output:$P_{Z}^{s,T}$**
*RandomSampling* (*Z*,*T*,*ξ*,*s*,*σ*,*p*,*sum*)
(01)*cnt* = 0
(02)**while** *cnt* < *maxCnt*
(03) *σ*←*s*
(04) *p*←*p*_0_
(05) **while** |*σ*| < *ξ* **and** *s* ∉ *T*
(06) **if** *next*(*s*, *σ*) ≠ Ø
(07) Select *s*_*i*_ from *next*(*s*, *σ*) with probability of *penNodeP*(*s*,*s*_*i*_,*σ*)
(08) *σ* ← *σ* ⋃ *s*_*i*_
(09) *p* ← *p* × *penNodeP*(*s*,*s*_*i*_)
(10) *s* ← *s*_*i*_
(11) **else**
(12) traceback *σ* to get a node *s*′ **satisfies** *next*(*s*′, *σ*) ≠ Ø
(13) Select *s*_*j*_ from *next*(*s*′, *σ*) with probability of *penNodeP*(*s*′,*s*_*j*_,*σ*)
(14) *path*←*path*⋃ *s*_*j*_
(15) *p* ← *p* × *penNodeP*(*s*′,*s*_*j*,_*σ*)
(16) *s* ← *s*_*j*_
(17) *sum*←*sum*+*p*
(18) *cnt* ++
(19)**return** *sum*

#### Simulated annealing algorithm

The DCD problem is a resource distribution problem which has |*DC*|×|*V*| decision variables and 2ǀ*DC*ǀ+1 constraints. This is difficult to solve using 0–1 programming because of the large number of variables. As an optimization algorithm, SA algorithm [45] is ideal to solve this type of problem. SA is a heuristic method for solving global optimization problems with a large solution space. It can escape from local optima and searches global optimal solution effectively. In addition, SA is also very simple and easy to implement. Therefore, SA is used to solve the DCD problem in this paper. The basic elements are defined as follows.

State expression: the binary-encoded vector is adopted as state expression. $x=\left\{x_{i}^{j}|i\in [1,\left|V\right|],j\in [1,\left|DC\right|]\right\}$ represents a state, where $x_{i}^{j}\in \{0,1\}$.

Objective function: SA algorithm is used to solve non-constrained optimization problems. To solve DCD, which is a constrained optimization problem, a punishment function is designed to transfer DCD to an unconstrained optimization problem, as shown in formula (13), where 3 penalty terms are introduced.

$$\begin{array}{c} \text{Min}\;E\left(x\right)=P_{Z^{+}}^{O,T}\left(x\right)+\left(\frac{\alpha }{T_{k}}\right)\sum_{i\in W}{\left(\sum_{j\in [1,\left|DC\right|]}l_{j}\times x_{i}^{j}-c_{i}\right)}^{2}\\ +\left(\frac{\beta }{T_{k}}\right)\sum_{i\in W^{\prime }}{\left(\sum_{j\in [1,\left|DC\right|]}x_{i}^{j}-h_{i}\right)}^{2}+\varphi \left(\frac{\gamma }{T_{k}}\right){\left(\sum_{i\in [1,V]}\sum_{j\in [1,\left|DC\right|]}x_{i}^{j}\times l_{j}-\theta \sum_{k\in [1,\left|V\right|]}h_{k}\right)}^{2} \end{array}$$ (13)

*α*, *β*, *γ* are the penalty factors, and *T*_*k*_ is the temperature parameter. *W* is the set of servers with overloaded capacity when *x* is deployed. *W*′ is the set of servers with overloaded decoy chains when *x* is deployed. *φ* is a Boolean variable, which equals 1 when the maximum capacity utilization rate exceeds *θ*. As the SA runs, *T*_*k*_ will decrease gradually. Accordingly, penalty terms will increase to ensure global searching at the beginning of SA and local searching at the end of SA. *E*(*x*) is defined as the energy at state *x*.

Neighborhood: The neighborhood of *x* is defined as *N*(*x*). Each element *x*′∊ *N*(*x*) is obtained by changing the value of an arbitrary $x_{i}^{j}\in x$ (*i* ∊[1,ǀ*V*ǀ], *j* ∊ [1, |*DC*|]). At temperature *T*_*k*_, the state transition probability from *x* to *x*′∊ *N*(*x*) is calculated with the Metropolis criterion [46], as shown in formula (14), where Δ*E* = *E*(*x*′) − *E*(*x*). Metropolis criterion models the state transition of a thermodynamic system. In this transition, the energy content is being minimized. Metropolis criterion enables SA to jump out of local optimum and achieve the global optimum solution.

$$P_{T_{k}}\left(x,x^{\prime }\right)=\{\begin{array}{ll} 1, & \text{if}\;E(x^{\prime })\leq E(x)\\ \text{exp}\left(-\frac{\Delta E}{T_{k}}\right), & \text{else} \end{array}$$ (14)

Cooling rule: Cooling rule is used to simulate cooling process, which grantees the convergence of SA and further assures the global optimum solution of DCD problem. In this paper, the selected cooling rule is shown in formula (15), which was proposed in literature [46]. *a* is a constant slightly smaller than 1, which determines the speed of cooling. The larger the value is, more slowly the temperature decreases. Empirically, *a* = 0.95. Thus, the temperature drops with a reasonable low speed and prevents SA from being trapped into a local optimum.

T(k+1)=a·T(k),k=0,1,2,⋯ (15)

The SA for DCD problem is designed as follows.

1. Initialize the parameters of SA. Choose an arbitrary initial solution *x*. Set annealing temperature as *T*_0_, end temperature as *T*_*f*_ and iterator as *k*. *T*_*k*_ = *T*_0_.
2. For the current solution *x*, generate a neighborhood solution *x*′∊ *N*(*x*). Calculate the increment of the energy value Δ*E* = *E*(*x*′) − *E*(*x*).
3. If Δ*E<* 0, set x = *x*′ and go to step 4, otherwise generate *μ* = *U*(0,1). If $\text{exp}\left(-\frac{\Delta E}{T_{k}}\right)<\mu$, set x = *x*′.
4. If thermal equilibrium is attained, go to step 5, otherwise go to step 2.
5. Decrease *T*_*k*_, *k* = *k* + 1. If *T*_*k*_ < *T*_*f*_, stop SA, otherwise go to step 2.

## Instructions for implementation and simulation experiments

### Instructions for implementation

**Fig 7:**
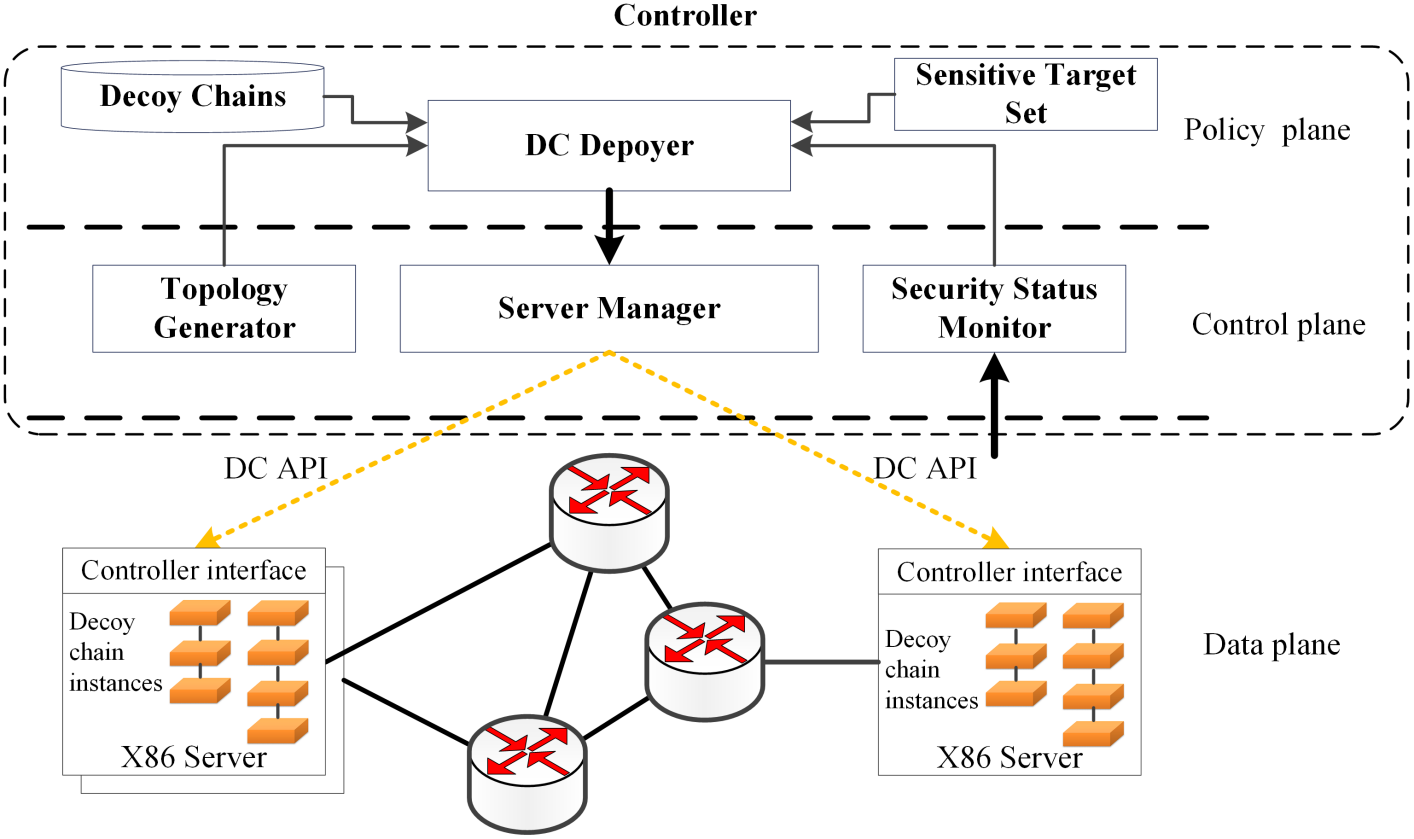
The schematic illustration of DCD system.

DCD is designed based on the architecture of SDN and NFV and one possible way to implement it is shown in Fig 7. There are three planes in this design: the policy plane, control plane and data plane. The policy plane and the control plane can be built based on the SDN controller. On the control plan, a Topology Generator can be adopted to provide network topology information. The Security Status Monitor can be used to monitor the network security status as well as potential attack source information. On the policy plane, Sensitive Target Set can be set based on users’ requires to provide sensitive targets information. With these essential information, a DC Deployer could be used to generate the decoy chain deployment strategies and deliver them to the Server Manager. At last, the decoy chain instances can be deployed in servers on the data plane through DC API, a southbound interface.

As the possible implementing scheme described above, the controller can control the data plane, collect security information from the network and quickly response to any network security threats. The controller is also able to directly control servers in the data plane and change the network attack surface by deploying the decoy chains in servers. When the network threat is removed, the controller will delete the decoy chain instances that have been deployed in the servers to decrease the server load and the resource consumption of the network. In order to reduce the latency of decoy chain deployment and economize bandwidth, the decoy nodes can be stored in the servers’ disks. When a server receives commands from the controller through DC API, the decoy chains can be constructed quickly. The latency of deployment will be sufficiently shortened and no traffic will be caused by transporting of virtual machines.

### Simulation experiments and evaluation

Penetration probability improvement (*PPI*) is introduced in this section to evaluate the effectiveness of DCD, as defined in formula (16). *Z* is the original penetration topology, while *Z*^+^ is the penetration topology deployed with decoy chains.

$$PPI=\left(1-\frac{P_{Z^{+}}^{O,T}}{P_{Z}^{O,T}}\right)\times 100\%$$ (16)

We implemented a DCD simulator with a C program with accordance to realistic networks, penetration attacks and decoy chain deployment. The DCD simulator complies with the basic nature and property of network, as well as realistic attack-defense interaction. In the experiments, BRITE [47], is used as the network topology generator. Four network random topologies are generated based on the Waxman model [47] with 16, 32, 48, 64 nodes (m = 2). All networks in our experiments have four layers, and each layer has the same number of nodes. Attack sources are in the outermost layer of the network, while sensitive targets are in the innermost layer. We assume that the *π* and *f* of each node in the network are 1. Every node in the network connects with a server. The capacity of these servers is denoted as *c*, which follows the distribution as shown in formula (17), where *F*(*x*) represents the probability distribution function of normal distribution with *μ* = *σ* = 5. The maximum load of decoy chains on server *h* follows the distribution as shown in formula (18), where *F*′(*x*) represents normal distribution with *μ* = 3, *σ* = 1.

$$P\left(c\right)=\frac{F\left(c+1\right)-F\left(c\right)}{F\left(16\right)-F\left(0\right)},0\leq c\leq 15$$ (17)

$$P^{\prime }\left(h\right)=\frac{F^{\prime }\left(h+1\right)-F^{\prime }\left(h\right)}{F^{\prime }\left(6\right)-F^{\prime }\left(0\right)},0\leq h\leq 5$$ (18)

Penalty factors *α*, *β*, *γ* are all set to 1 in the experiments. Ten kinds of decoy chains with lengths ranging from 1~10 are used in the experiments. The maximal capacity usage rate *θ* is set to 0.5. The DCD simulator runs on a 64 bit computing platform with 2.53 GHz Intel Xeon CPU and 32G RAM.

#### Validation of the effectiveness of DCD

**Fig 8:**
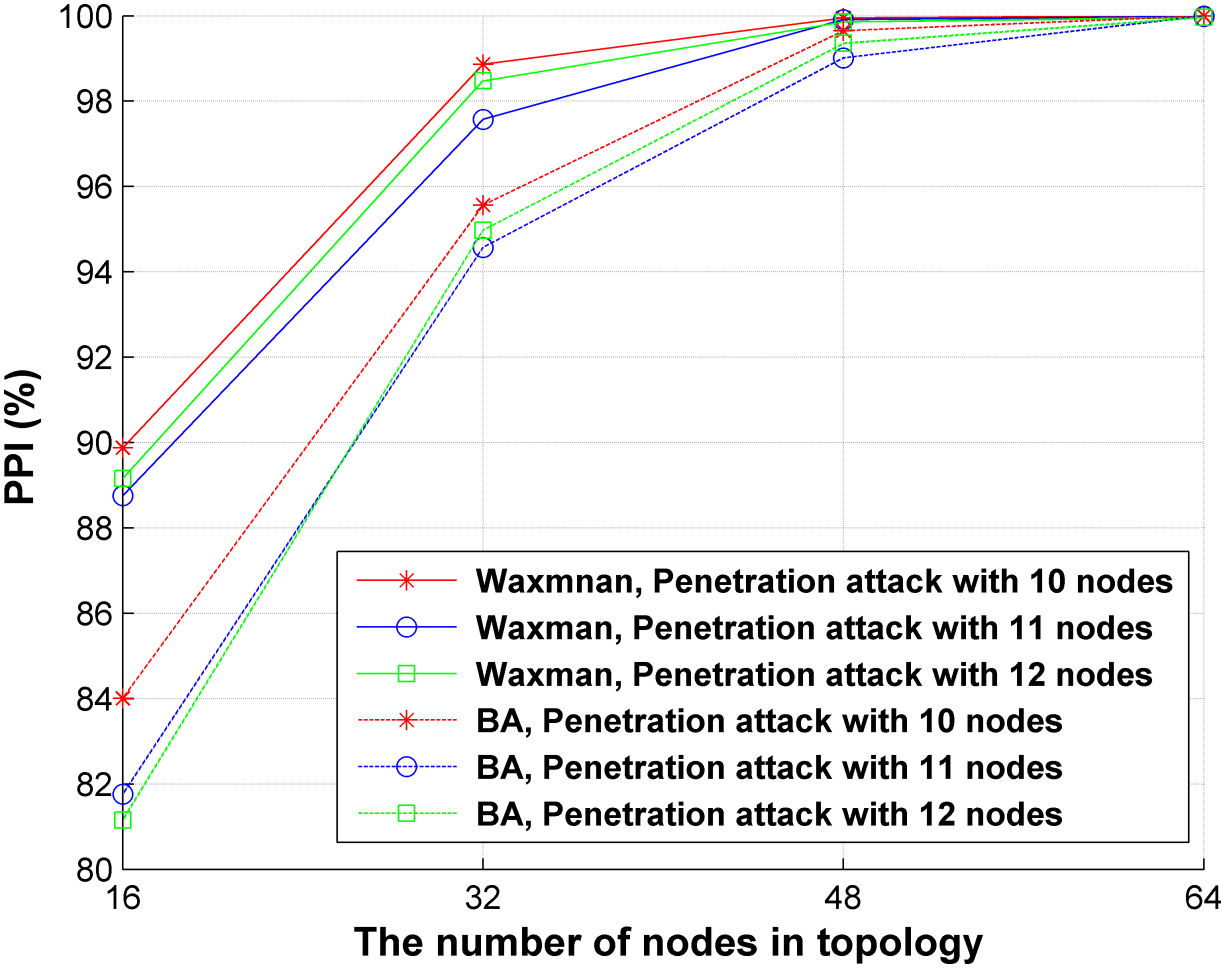
Comparison of *PPI* with different topologies and *ξ*.

In this experiment, decoy chains are deployed using DCD in the four generated networks. We also generated four networks based on the BA model [48] using BRITE for comparing the effects of topologies on our method. One attack source and one sensitive target are set in these networks. The maximum length of the penetration path *ξ* varies between 10~12. The penetration probability is computed using *RandomSampling* (*maxCnt* = 10^4^). Multiple tests are conducted, and the average result is computed to eliminate the uncertainty brought by randomness. A series of *PPI* is obtained with different topologies and penetration path lengths, as shown in Fig 8.

As seen, *PPIs* are larger than 80% for all cases, which indicates that the probability of sensitive targets being penetrated by intruders decreased after deploying decoy chains. It also shows that the larger the network size is, the higher the *PPI* can be achieved, resulting in stronger resistance to penetration attacks. The reason is that more decoy chains can be deployed on larger networks, making them more complicated to be penetrated. It can be found that DCD could get similar results on the Waxman model network and BA model network. Therefore, DCD is effective on networks of different models.

**Fig 9:**
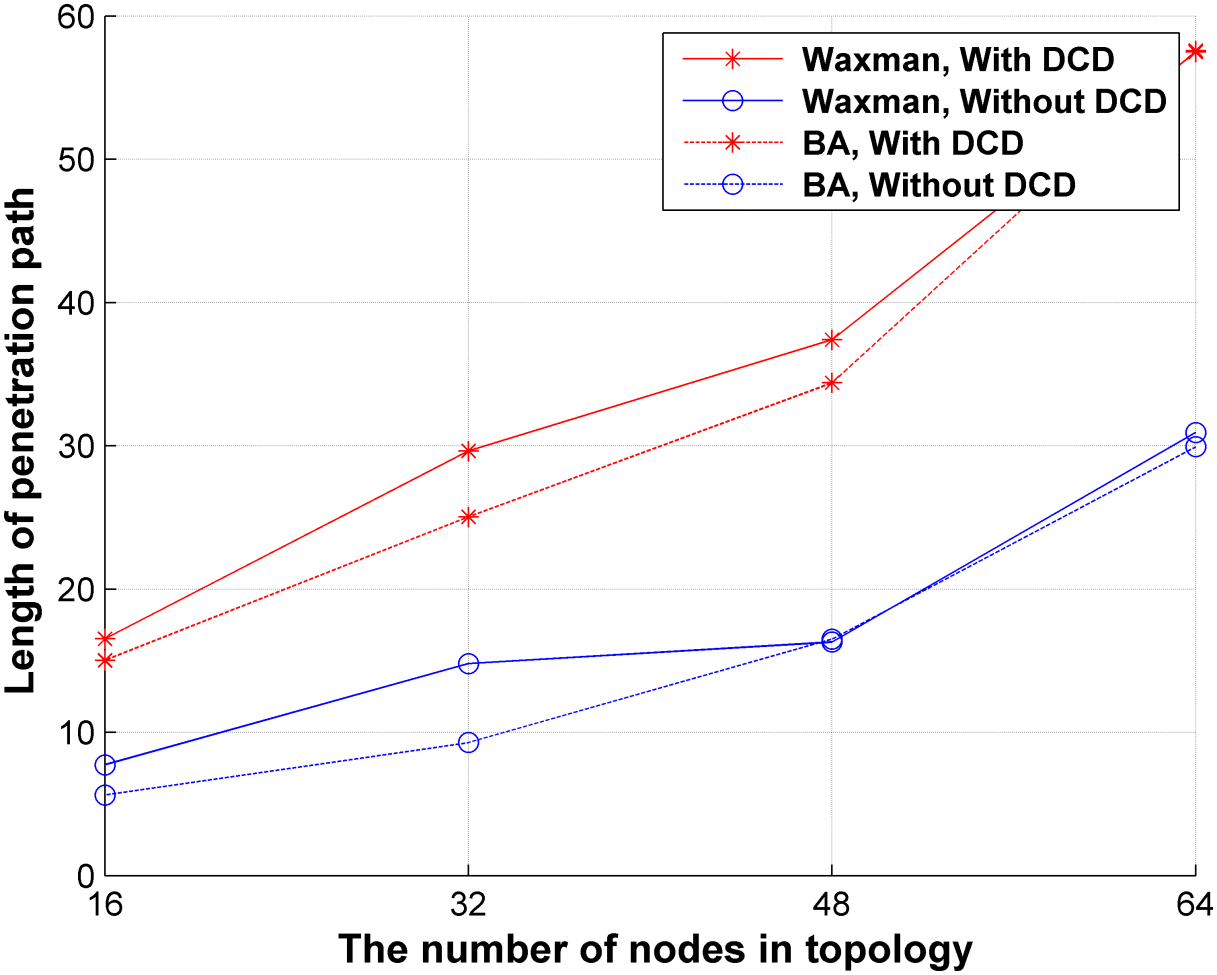
Comparison of the length of penetration paths with and without DCD.

Decoy chains are deployed to simulate parts of the network. It is difficult for intruders to distinguish real nodes from decoy nodes when they try to penetrate the network. Assume that an intruder penetrates the network using the random-walk method: he starts from an attack source and selects an adjacent node according to the probability calculated by formula (6). If no adjacent nodes can be selected, he will trace back and continue to select nodes to penetrate until a sensitive target is compromised. The average lengths of penetration paths that compromise sensitive targets in both Waxman networks and BA networks with and without DCD are compared, and results are shown in Fig 9. As we can see, the average length of the penetration path with DCD is 1.069 times greater than that without DCD. A greater length in the penetration path indicates increased attack time cost. The reason is that intruders easily falls into the trap of the decoy chains when they penetrate the network with DCD. The results of DCD upon the two network models are very similar, which means our method is robust to different networks.

#### Comparison between recursive traversal and random sampling algorithm

**Fig 10:**
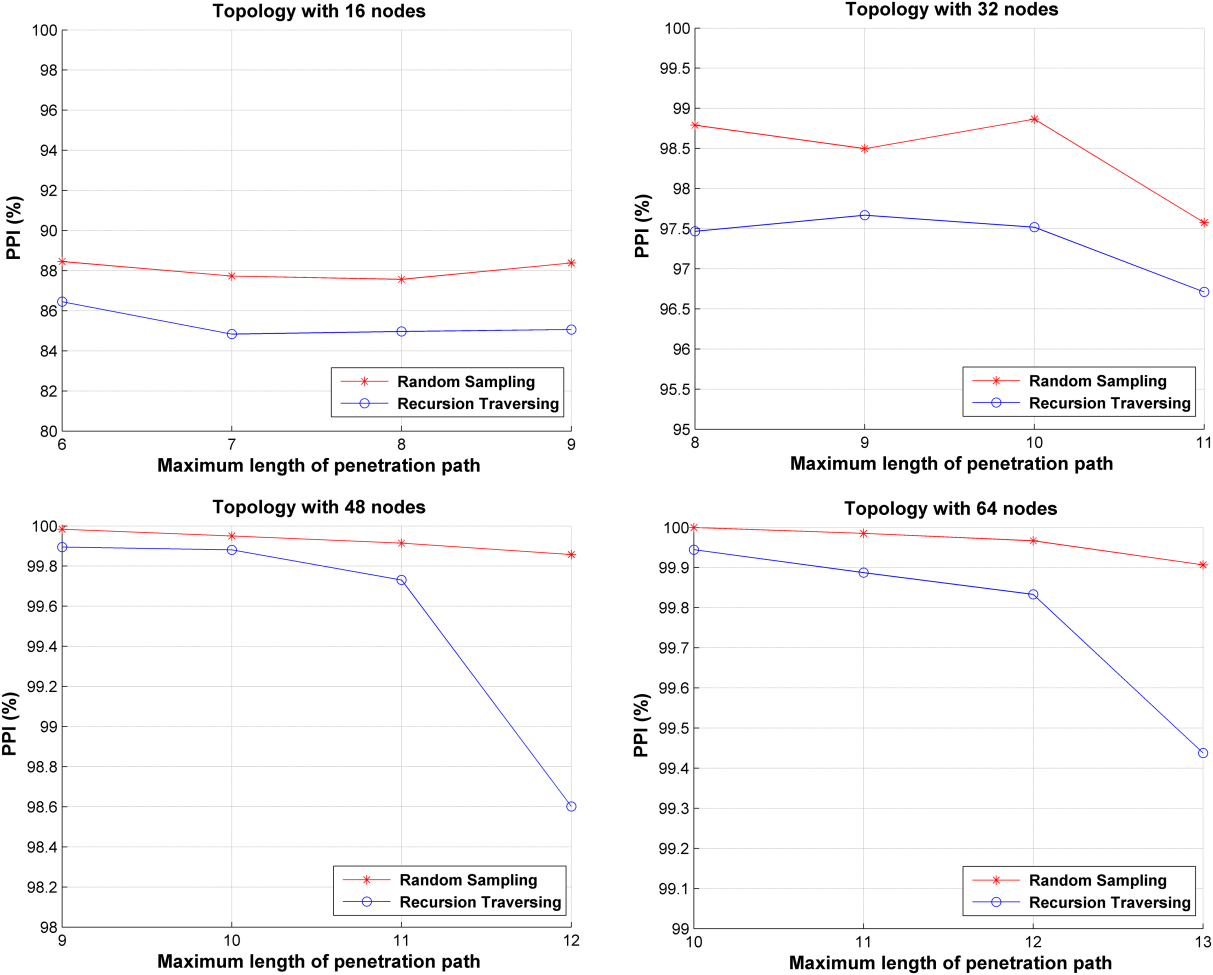
Comparison of *PPI* using recursive traversal and random sampling algorithm.

A series of experiments are conducted using the generated network topologies to compare the influence on DCD when recursive traversal and random sampling are used. In the experiments, the two algorithms are used to calculate energy in SA, and they generate decoy chain deployment strategies *x*^*recursion*^ and *x*^*sample*^, respectively. In this experiment, 10^4^ penetration attacks are launched using random-walk to the network with one of the two strategies. The comparison of *PPI* is shown in Fig 10. The horizontal axis represents *ξ* and the vertical axis represents *PPI*. These results show that the random sampling method can achieve a higher *PPI* in all topologies. This is because the deployment of the decoy chains using the recursive traversal method aims at all penetration paths. On the contrary, the random sampling method tends to deploy decoy chains for the paths that have higher penetration probabilities. Furthermore, these paths with higher penetration can be more likely to be penetrated using random-walking. Therefore, the SA using random sampling better suits the DCD problem and achieves higher *PPI* rate.

**Fig 11:**
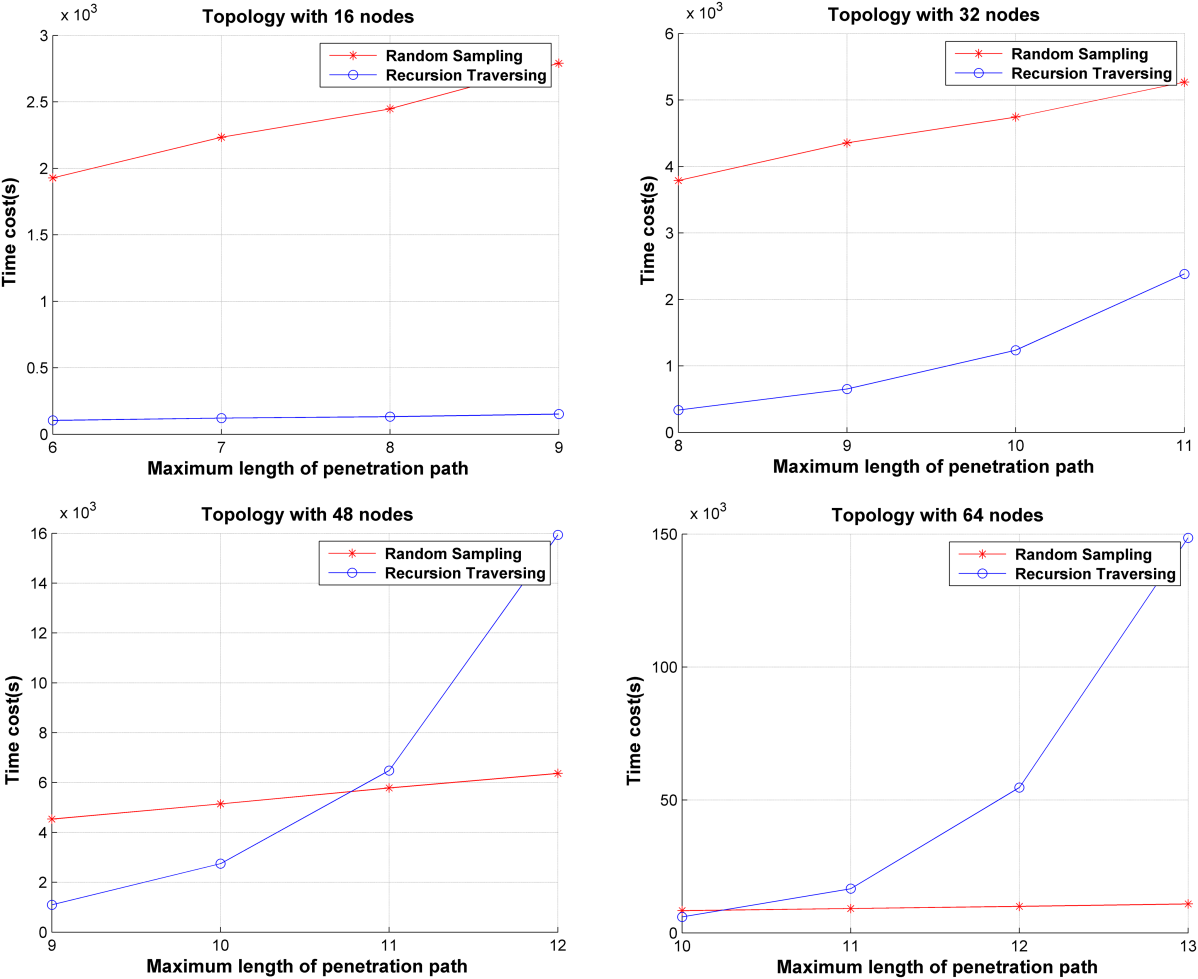
Comparison of time cost between SA with recursive traversal and random sampling algorithm.

The time costs of SA using the recursive traversal algorithm and random sampling algorithm are compared under several generated network topologies. In the random sampling algorithm, we set *maxCnt* to 10^4^ and the time costs are shown in Fig 11. It can be concluded that SA using recursive traversal algorithm is more efficient than using random sampling algorithm when the scale of the network is small. The reason is obvious: there are fewer penetration paths in a small-scale network, and those paths can be traversed with less time cost. As the network scale and the maximum length of the penetration path grow, the number of possible penetration paths increases exponentially. Therefore, the time cost of SA using the recursive traversal algorithm increases exponentially. However, the growth of the time cost is much less when the random sampling algorithm is used, as the number of penetration paths remain unchanged. Therefore, the random sampling algorithm can get a lower time cost in larger-scale networks.

#### Comparison of greedy algorithm and DCD

**Fig 12:**
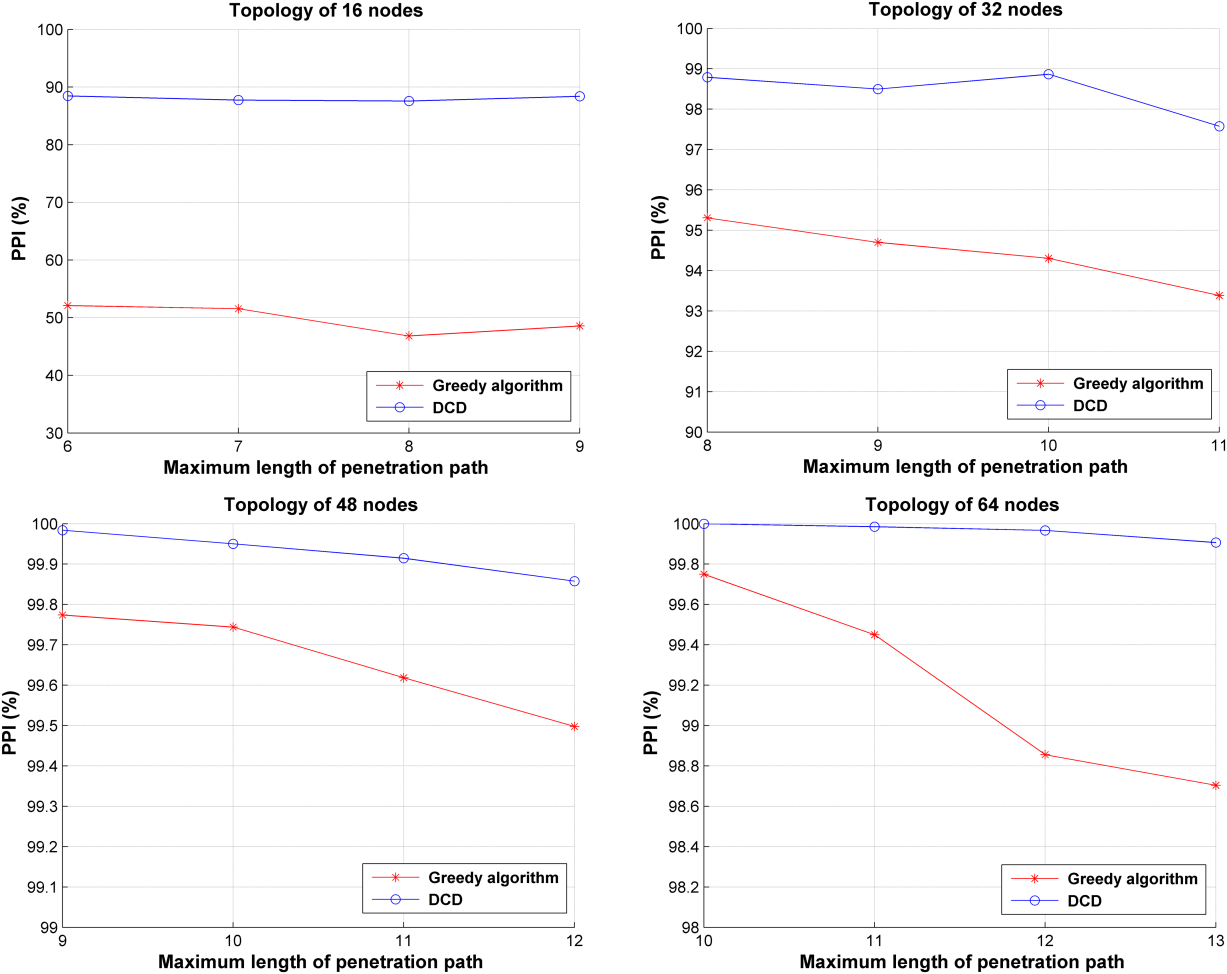
Comparison between greedy algorithm and DCD.

According to literature [14], it is helpful to reduce the penetration probability when the decoy chains are deployed on nodes with small degrees. Therefore, we compared the effectiveness of DCD and greedy algorithm. The greedy algorithm deploys decoy chains as many as possible to the nodes with the smallest degrees under the resource constraints. The comparison between the greedy algorithm and DCD is made in experiments with the generated network topologies. We set *maxCnt* to 10^4^ in this experiment and the *PPIs* are shown in Fig 12. As seen, the greedy algorithm can reduce the penetration probability (*PPI > 0*), which is consistent with literature [14]. However, it provides much lower security compared with DCD. DCD tries to find the global optimum solution, while the greedy algorithm can only give a local optimal solution. Therefore, DCD can provide better security by reducing probability of sensitive targets being compromised in a greater degree.

#### DCD with multiple attack sources and targets

DCD is able to deploy decoy chains for multiple attack sources and sensitive targets. The generated network with 64 nodes is used in this experiment. Two nodes are randomly chosen from the first layer of the network as the attack source set *O* = {*o*_1_,*o*_2_} and two nodes from the fourth layer constitute the sensitive target set *T* = {*t*_1_,*t*_2_}. The intruder appears at *o*_1_ and *o*_1_ with equal probability and we set *ξ* = 12. Random sampling algorithm (*maxCnt* = 10^4^) is used to calculate the penetration probability $P_{Z}^{\left\{o_{i}\}\to \{t_{j}\right\}}$ (*i*, *j* ∊ {1, 2}). The relative probability *RP*_*ij*_ is defined as formula (19).

$$RP_{ij}=\frac{P_{Z}^{\left\{o_{i}\}\to \{t_{j}\right\}}}{\text{max}\left\{P_{Z}^{\left\{o_{h}\}\to \{t_{k}\right\}}|o_{h}\in O,t_{k}\in T\right\}},\;i\in \left[0,\left|O\right|\right],j\in \left[0,\left|T\right|\right]$$ (19)

**Fig 13:**
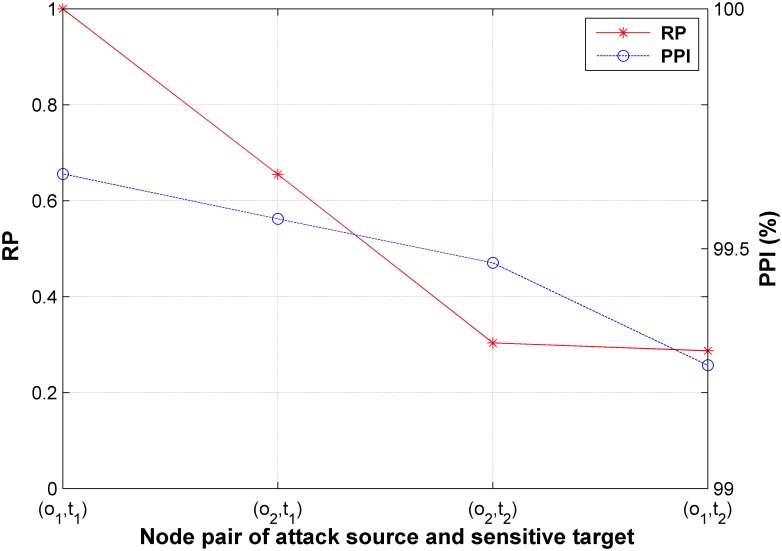
Deployment of the decoy chains with multiple attack sources and sensitive targets.

The *RP*_*ij*_ of the each OT pair (*o*_i_, *t*_j_) (*o*_i_ ∊ *O*, *t*_j_ ∊ *T*), is obtained in the experiment, as shown by the solid line in Fig 13. Then, the decoy chains are deployed in the network using DCD. The *PPI* of each pair (*o*_i_, *t*_j_) is shown by the dashed line in Fig 13. The result shows that OT pairs with higher *RPs* achieve higher*PPIs*, meaning that a better security is achieved by DCD for an OT pair with higher *RP*. DCD considers the global optimation for deploying decoy chains. Thus, the OT pairs with the higher *RPs* are considered preferentially. Therefore, an OT pair with higher *RP* achieves a higher *PPI* after deploying decoy chains.

## Discussion

We view DCD as a promising method to defense penetration attacks. As the simulation experiments shown, DCD can effectively decrease the probability of sensitive targets being compromised. We believe that DCD has a great potential in improving the security of network, even though the performance is not ideal. Further optimization could greatly facilitate its application.

In this study, we concentrate on outside intruders that appear at the edge of network. Accordingly, the decoy chains are deployed for these outside intruders. However, the insider intruders who appear at inner layers of the network may bypass several layers of the network. Therefore, their penetration actions may not be effectively defensed by the current DCD. The defense of inside attack will be further studied in future work.

In the experiments, the time cost for generating decoy chain deployment strategy is not ideal. The reasons are: 1) In the experiments, we choose a big value for the number of random samples of paths (*maxCnt* = 10^4^) to adequately test the security of DCD, which leads to large time cost of random sampling algorithm. 2) Both the design and implementation of DCD are preliminary and can be optimized using parallelism techniques. Moreover, the performance could be further improved by executing part of the operation offline, instead of online, even though DCD is designed for responding network security threats online. More specifically, decoy chain deployment strategies could be calculated in advance, since the attack sources (terminals with poor protection) and sensitive targets (terminals with sensitive data) do not change frequently. Furthermore, for deploying decoy chain, if DCD sends the whole decoys to the servers, both the time cost and bandwidth cost will be large. Considering the availability of DCD, the decoy nodes can be stored in disks of the servers in advance. When a server receives commands from the controller, the decoy chains can be constructed at once so that the latency could be greatly shortened.

In this paper, the capacity of a server is defined to represent the maximum number of virtual machines running on the server. However, the capacity of a server is a coarse-grained resource constraint and a more fine-grained approach could be considered. One possible way would be calculating multiple resource constraints respectively, such as the number of CPU, memory size, hard disk driver, and bandwidth. However, the fine-grained resource constraints will make the decoy chain model much more complex.

## Conclusion and future work

In this paper, MTD is introduced to deploy the decoy chains in the network for changing the attack surface. DCD, a decoy chain deployment method, is proposed based on SDN and NFV. DCD creates a second defense line for the network, which can delay the penetration attack and decrease the probability that sensitive targets are compromised. Centralized control of SDN is utilized to deploy decoy chains under resource constraints, and multiple attack sources and sensitive targets are considered. SA algorithm is used in DCD to achieve the maximum benefit for network defense. Experiments show that DCD can effectively defend against penetration attack. Future improvements could be achieved in three aspects as follows: 1) How to reduce the time cost of DCD; 2) How to deploy chains dynamically based on security monitoring to increase the efficiency of DCD; 3) In this paper, the decoy chain composing of decoy nodes has no branch. Therefore, it is easy to arouse intruders’ suspicions. Instead of the decoy chain, decoy-net, a network consists of decoy nodes, will also be explored in future work.
